# Supplementary material for: Learning to Approximate Functions Using Nb-Doped SrTiO3 Memristors
Source: Front Neurosci. 2021 Feb 19;14:627276. doi: 10.3389/fnins.2020.627276 (PMC7933504; doi:10.3389/fnins.2020.627276)
Supplement: Supplementary file 1 [file Data_Sheet_1.PDF]

frontiers

Supplementary Material

1 SUPPLEMENTARY DATA

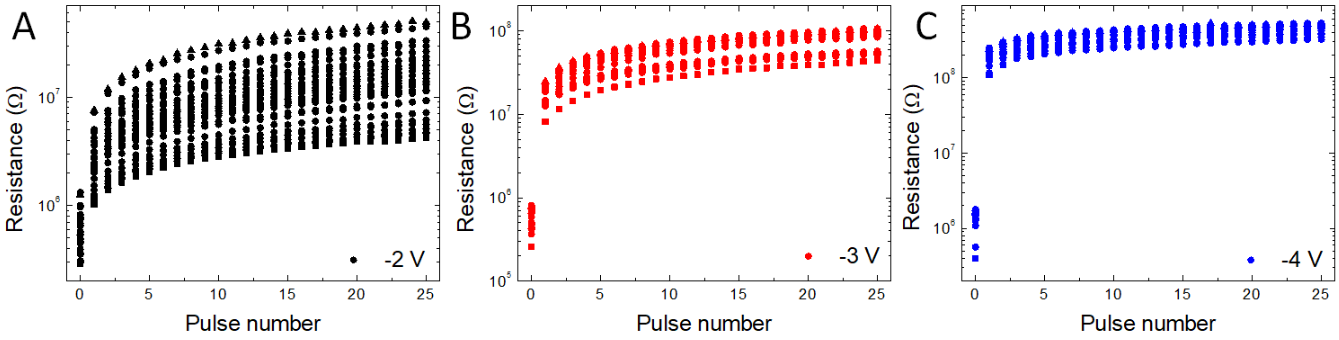

Figure S1. Device response to RESET pulses of **(A)** -2 V, **(B)** -3 V and **(C)** -4 V (also shown in main text). Variation in the initial state is reflected by differences in the resistance at pulse number 0. In all cases, the first pulse induces the largest change and the effect of subsequent pulses is diminished.

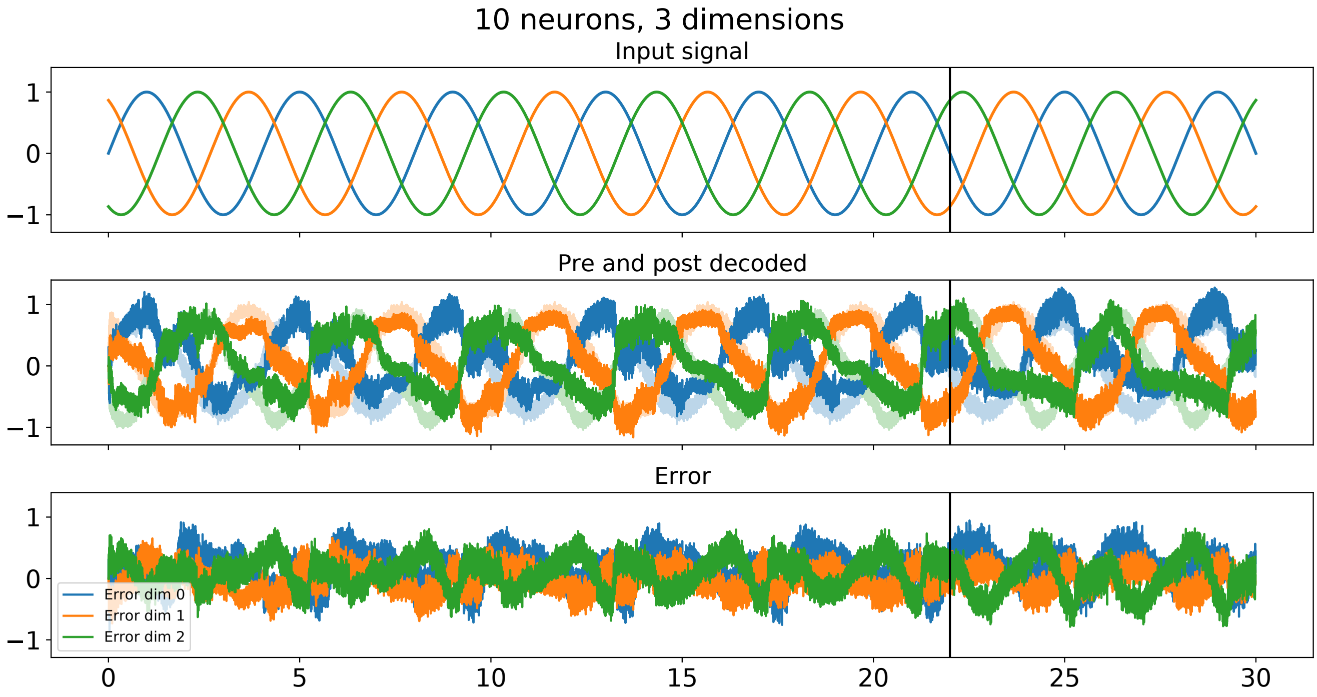

Figure S2. Example simulation during which a network composed of 10 pre- and post-synaptic neurons learns the identity function from a 3-dimensional sine wave. After 22 seconds learning is switched off (vertical black bar) and the performance is tested on the same sine wave. The top panel shows the input signal afferent to the pre-synaptic neuronal ensemble. The middle panel shows the decoded output from the pre=synaptic ensemble (faded colours), and from the post-synaptic ensemble (bold colours). The bottom panel shows the decoded output from the ensemble calculating the global error $\boldsymbol{E}$, which is used to drive learning.

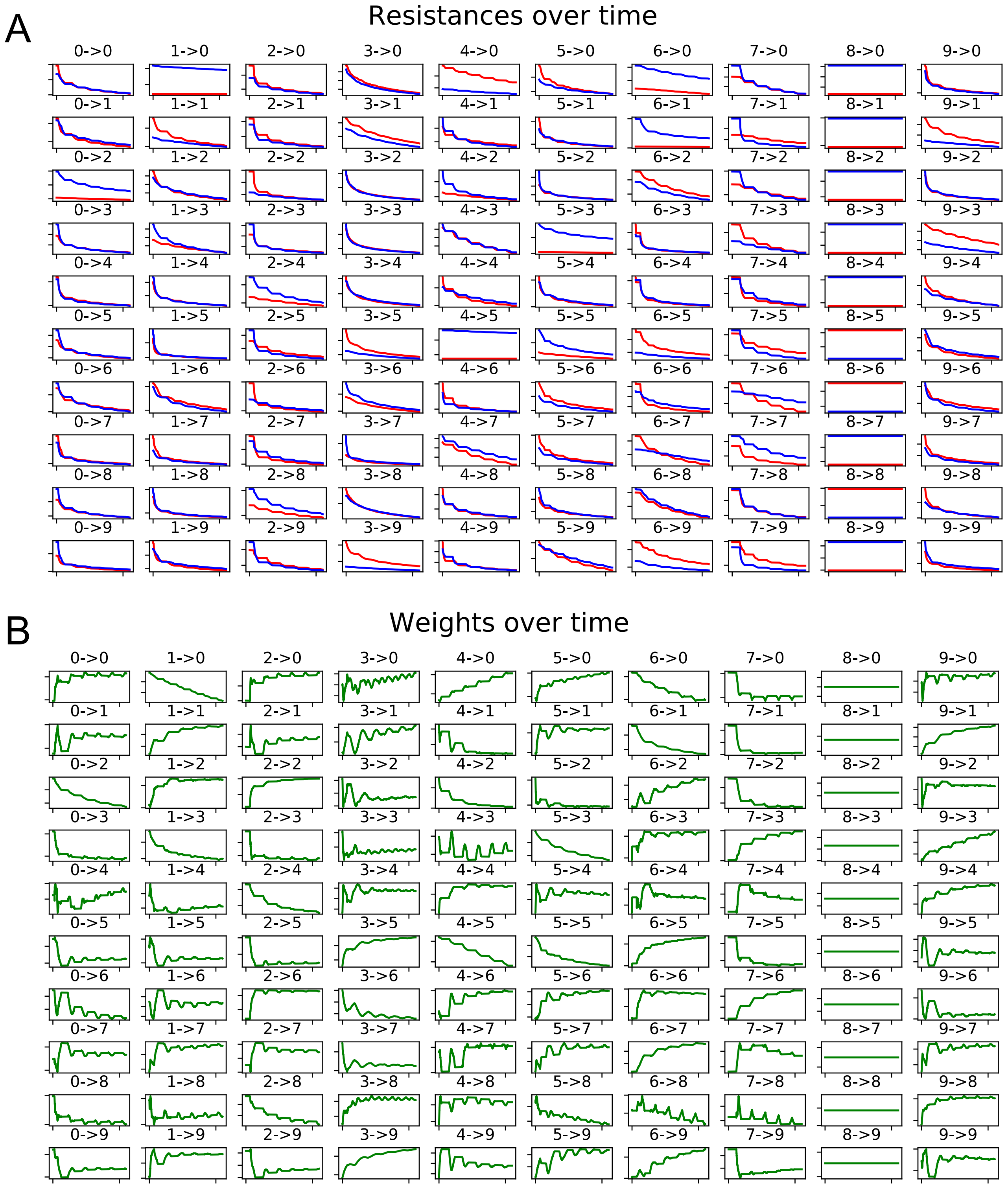

Figure S3. **(A)** Evolution of memristor resistances during the training phase for the network simulated in Fig. S2. The plots are organised such that the synapses efferent from pre-synaptic neuron i are in column i, and the synapses afferent to post-synaptic neuron j are in row j. Each plot shows the positive (in red) M_{ij}^{+} and negative (in blue) M_{ij}^{+} memristor for the synapse connecting pre-synaptic neuron i to post-synaptic neuron j. **(B)** Evolution of the network weights W_{ij} for each synapse connecting pre-synaptic neuron i and post-synaptic neuron j. Each each weight is given by $W_{ij} = \zeta(M_{ij}^{+} - M_{ij}^{-})$, with ζ a transformation of the memristor resistances to network weights.

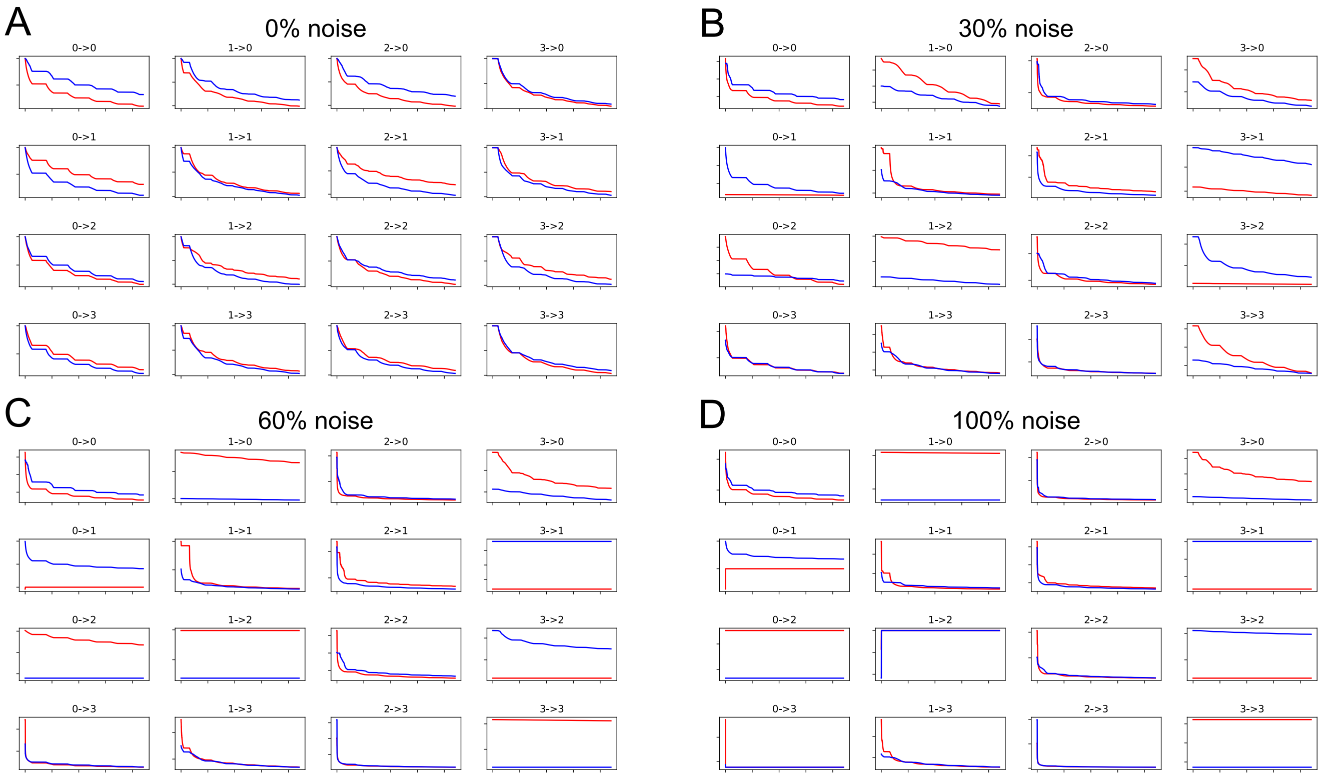

Figure S4. Evolution of memristor resistances during training for varying levels of coefficient of variation (expressed as percentage of noise) on the parameters R_0, R_1, c in the power-law governing the resistance update, and initial resistances. The variation was introduced via Gaussian random sampling. The network for this example was run with 4 pre- and post-synaptic neurons. **(A)** No noise added to parameters so each memristor in the network has the same update behaviour. All memristors start from the same HRS of $10^8\,\Omega$. **(B)** 30% of coefficient of variation added to all parameters. The memristors start from an initial resistance $[10^8\,\Omega \pm 30\%]$ and vary in their response to learning SET pulses. **(C)** 60% of coefficient of variation added to all parameters. Some memristors don't respond to pulses because their initial resistance was already equal to the minimum R_0, or to the maximum R_1. **(D)** 100% of coefficient of variation added to all parameters. Many memristors don't respond, or stop responding because their resistance quickly saturates. Some memristors see their resistance increase in response to SET pulses.
